# Alzheimer's disease neuropathology and its estimation with fluid and imaging biomarkers

**DOI:** 10.1186/s13024-025-00819-y

**Published:** 2025-03-14

**Authors:** Dietmar Rudolf Thal, Koen Poesen, Rik Vandenberghe, Steffi De Meyer

**Affiliations:** 1 Department of Imaging and Pathology, Laboratory for Neuropathology, Leuven Brain Institute, KU Leuven, Herestraat 49, Leuven, 3000 Belgium; 2 Department of Pathology, University Hospitals Leuven, Leuven, Belgium; 3 Department of Neurosciences, Laboratory for Molecular Neurobiomarker Research, Leuven Brain Institute, KU Leuven, Leuven, Belgium; 4 Department of Laboratory Medicine, University Hospitals Leuven, Leuven, Belgium; 5 Department of Neurosciences, Laboratory for Cognitive Neurology, Leuven Brain Institute, KU Leuven, Leuven, Belgium; 6 Department of Neurology, University Hospitals Leuven, Leuven, Belgium

**Keywords:** Alzheimer's disease, Tau, Amyloid, TDP-43, α-synuclein, Neuropathology, Imaging, Fluid biomarkers

## Abstract

Alzheimer’s disease (AD) is neuropathologically characterized by the extracellular deposition of the amyloid-β peptide (Aβ) and the intraneuronal accumulation of abnormal phosphorylated tau (τ)-protein (p-τ). Most frequently, these hallmark lesions are accompanied by other co-pathologies in the brain that may contribute to cognitive impairment, such as vascular lesions, intraneuronal accumulation of phosphorylated transactive-response DNA-binding protein 43 (TDP-43), and/or α-synuclein (αSyn) aggregates. To estimate the extent of these AD and co-pathologies in patients, several biomarkers have been developed. Specific tracers target and visualize Aβ plaques, p-τ and αSyn pathology or inflammation by positron emission tomography. In addition to these imaging biomarkers, cerebrospinal fluid, and blood-based biomarker assays reflecting AD-specific or non-specific processes are either already in clinical use or in development. In this review, we will introduce the pathological lesions of the AD brain, the related biomarkers, and discuss to what extent the respective biomarkers estimate the pathology determined at post-mortem histopathological analysis. It became evident that initial stages of Aβ plaque and p-τ pathology are not detected with the currently available biomarkers. Interestingly, p-τ pathology precedes Aβ deposition, especially in the beginning of the disease when biomarkers are unable to detect it. Later, Aβ takes the lead and accelerates p-τ pathology, fitting well with the known evolution of biomarker measures over time. Some co-pathologies still lack clinically established biomarkers today, such as TDP-43 pathology or cortical microinfarcts. In summary, specific biomarkers for AD-related pathologies allow accurate clinical diagnosis of AD based on pathobiological parameters. Although current biomarkers are excellent measures for the respective pathologies, they fail to detect initial stages of the disease for which post-mortem analysis of the brain is still required. Accordingly, neuropathological studies remain essential to understand disease development especially in early stages. Moreover, there is an urgent need for biomarkers reflecting co-pathologies, such as limbic predominant, age-related TDP-43 encephalopathy-related pathology, which is known to modify the disease by interacting with p-τ. Novel biomarker approaches such as extracellular vesicle-based assays and cryptic RNA/peptides may help to better detect these co-pathologies in the future.

**Supplementary Information:**

The online version contains supplementary material available at 10.1186/s13024-025-00819-y.

## Background

Alzheimer's disease (AD) is histopathologically characterized by the deposition of the amyloid-β peptide (Aβ) [1] as well as by the generation of intraneuronal neurofibrillary tangles (NFTs) consisting of abnormally phosphorylated tau (τ) protein (p-τ) [2]. To detect these hallmark lesions, neuropathological examination of tissue either from post-mortem brain autopsy or from ante-mortem brain biopsies is required [3]. Ante-mortem brain biopsies are currently not indicated for the in vivo diagnosis of AD because its result would not yield actionable clinical consequences. Therapeutic options are currently only provided to AD patients who exhibit at least mild symptoms. Treatment of asymptomatic patients can today only be considered in the context of clinical trials [4, 5]. Moreover, the impact of the currently available drugs on the course of AD is still limited [5, 6].

To allow an in vivo diagnosis of AD supported by biological evidence rather than solely clinical criteria, biomarkers have been developed to estimate the presence and severity of Aβ and p-τ pathology in patients ante-mortem. These biomarkers include positron emission tomography (PET) methods visualizing Aβ or p-τ in the brain with radiolabeled tracers that bind specifically to Aβ and p-τ aggregates [7–11] as well as measurement of Aβ or p-τ levels in cerebrospinal fluid (CSF) [12, 13] and blood [14, 15]. With these biomarkers it is now feasible to detect AD not only in symptomatic patients, but also in asymptomatic, also called preclinical, stages of the disease [16–18]. Moreover, imaging as well as fluid biomarkers have been developed that reflect downstream consequences of Aβ and p-τ pathology, including neuroinflammation and neurodegeneration, which are involved in AD pathophysiology but are not exclusive to AD. In addition to Aβ and p-τ pathology, most AD cases exhibit additional pathologies in the brain that may contribute to neurodegeneration, neuroinflammation and cognitive decline. Such co-pathologies are (1) the intraneuronal accumulation of phosphorylated transactive-response DNA-binding protein 43 (pTDP-43) [19–24], (2) the accumulation of α-synuclein (αSyn) inclusions in neurons [19, 23, 25, 26] and (3) vascular lesions [27–30].

In this review article, we aim to clarify which neuropathologically-defined AD hallmark lesions can be detected with the current biomarkers and at what stages of the disease they become detectable. Second, we will highlight common co-pathologies identified by neuropathologists in AD patients and evaluate whether biomarkers can trace the presence or absence of these co-pathologies. Moreover, novel biomarker approaches will be discussed. Finally, we will illustrate possible concepts of using current and upcoming biomarkers for future clinical diagnosis and clinical trial-related stratifications as well as concepts on interpreting biomarker-based pathogenetic studies.

## Neuropathological hallmark lesions

**Fig. 1:**
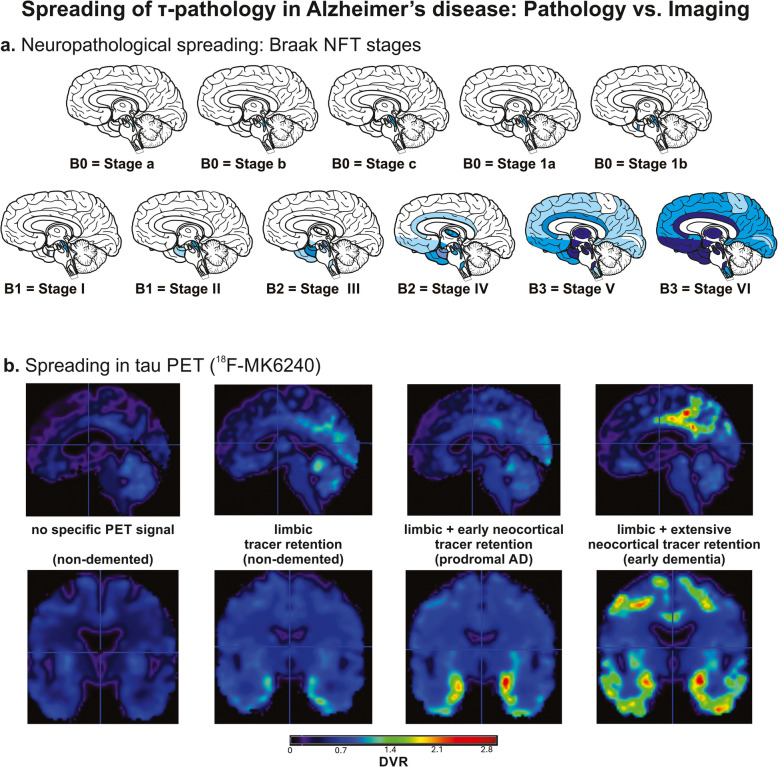
Spreading of p-τ pathology in the human brain. **a** The first neurons exhibiting p-τ are detected in the brain stem in the locus coeruleus, raphe nuclei, and neurons of the basal forebrain [38, 40–42]. When p-τ pathology converts into argyrophilic NFT pathology, the transentorhinal and entorhinal cortex becomes involved, followed by limbic regions, the basal neocortex until the entire cortex is filled with NFT pathology including the primary cortical areas. This process is reflected by the Braak NFT-stages [38, 43]. Note that only the “argyrophilic” Braak NFT stages are covered in the neuropathological recommendations for the diagnosis of AD [44] as indicated by the B-scores B1-B3. Brain stem Braak NFT stages are considered as B0. **b** τ PET imaging with the ^18^F-MK6240 tracer reflects this process and shows also a stage like propagation of tracer positive regions starting in the (trans)entorhinal cortex, spreading to the amygdala and adjacent temporobasal cortex and finally into the neocortex [45]. Tracer retention is provided as distribution volume ratio (DVR). This is demonstrated in four ^18^F-MK6240 τ PET images from (1) a 79-year-old, cognitively normal control case (CDR-score = 0) without tracer retention, (2) a 68-year-old, non-demented, asymptomatic AD case (CDR-score = 0) with initial entorhinal τ tracer retention, (3) a 68 year old prodromal AD case (CDR-score = 0.5) with mild cognitive impairment and tracer retention extending to adjacent medial temporal lobe regions including amygdala and temporal neocortex, and (4) a 77-year-old symptomatic AD case (CDR-score = 1) with extensive neocortical τ tracer retention. The 18F-MK6240 τ tracer was chosen for depicting pathology progression in τ PET because it is a second generation τ tracer that does not show significant off-target binding especially in the medial temporal lobe. Schematic representations in **a** are modified from Thal & Tomé 2022 [46] with permission

Since the first description of AD by Alois Alzheimer, NFTs and amyloid plaques have been indicated as the two neuropathological hallmark lesions [31]. NFTs were shown to represent fibrillar aggregates of the p-τ protein [32] whereas amyloid plaques mainly consist of fibrillar aggregates of Aβ [1]. Although Aβ_1–40_ is the most abundantly produced Aβ isoform, Aβ_1–42_ is the main constituent of extracellular amyloid plaques due to its propensity to aggregate [33, 34]. The Aβ peptide was also found to be the main constituent of vascular amyloid aggregates in cases with sporadic and some familiar forms of cerebral amyloid angiopathy (CAA) [35].

p-τ pathology in NFTs is accompanied by neuropil threads (i.e., p-τ accumulation in neurites) and pretangles (i.e., non-fibrillar accumulation of p-τ in the perikaryon of neurons) [36]. Precursor lesions in the form of initial cytoplasmic and neuropil τ have been described but it is not yet clear whether these lesions represent a still physiological, reversible status of τ phosphorylation or already indicate the start of an irreversible pathological process [37]. The evolution of cerebral p-τ pathology starts in the brain stem and extends into the transentorhinal (Braak NFT stage I), entorhinal cortex (Braak NFT stage II), limbic brain regions such as the hippocampus and the amygdala, the perirhinal cortex (Braak NFT stage III), the superior temporal (Braak NFT stage IV), tertiary and secondary neocortex (Braak NFT stage V), and ultimately primary cortical areas such as the primary visual cortex (Braak NFT stage VI) (Fig. 1) [38]. In this context, it is important to know that the development of p-τ pathology from Braak NFT stage I to VI can take 20 to 30 years [39]. Moreover, p-τ pathology is prevalent in nearly all individuals older than 40 years of age when considering brain stem p-τ pathology as the starting point [38].

**Fig. 2:**
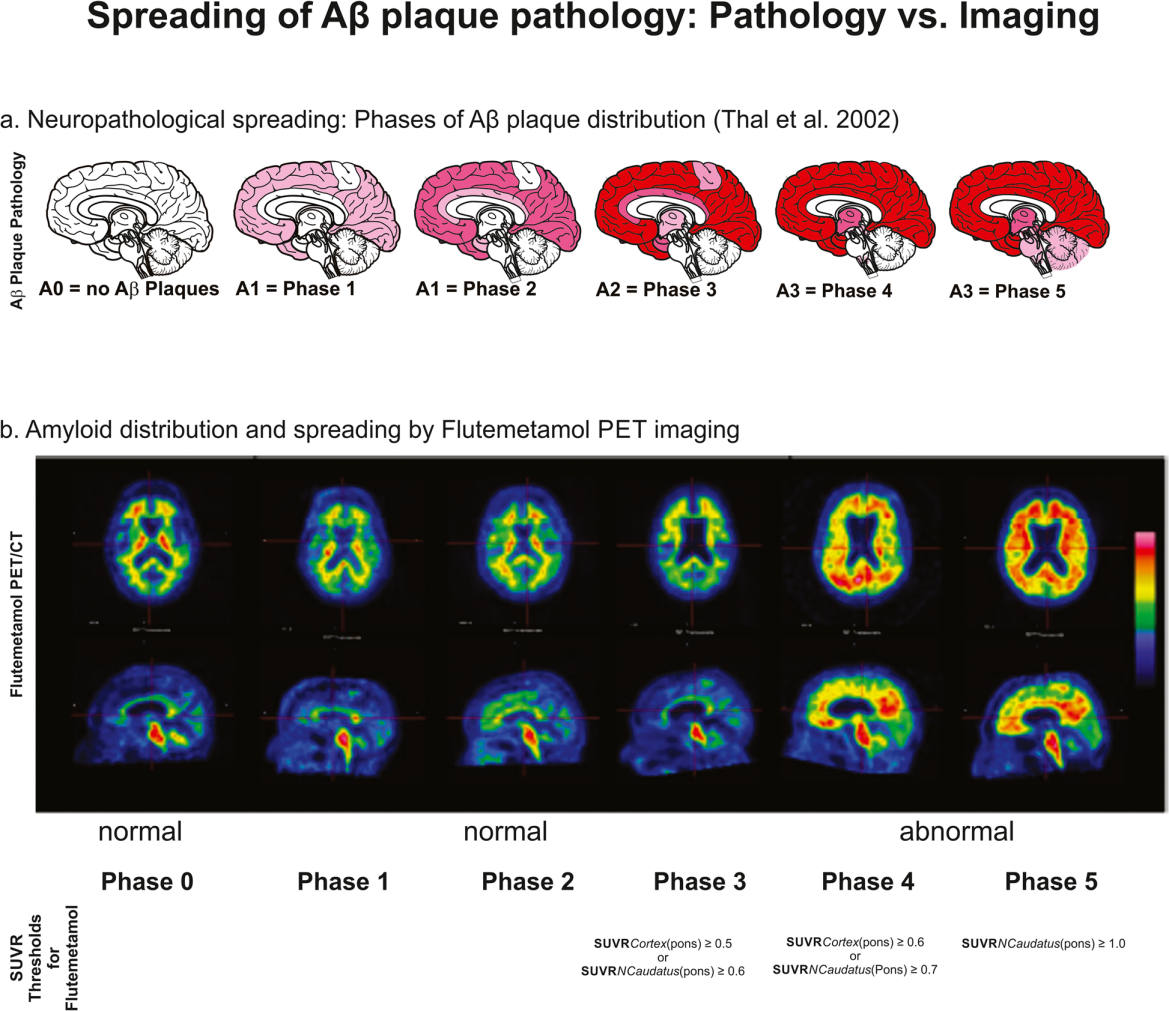
**a** Spreading of Aβ plaque pathology. Neuropathologically, the first plaques are found in the neocortex. The light-pink area describes, in a given phase, the area that the neuropathologist has to screen to find the first 2–3 amyloid plaques. Most of the area can be free of plaques at this stage. Full pink marked areas represent usually moderate amounts of plaques whereas full red marked areas show full-blown plaque pathology. The first area affected is the neocortex in phase 1. Propagation into allocortex (phase 2), basal ganglia & diencephalon (phase 3), midbrain & medulla oblongata (phase 4), and finally into pons & cerebellum in phase 5 is indicated by the respective Aβ phases and the A (amyloid)-scores [44, 55]. **b**^18^F-Flutemetamol amyloid PET images indicate that in Aβ phases 1–2 tracer retention does not obviously differ from Aβ phase 0 cases whereas Aβ phases 4 and 5 are easily identifiable by eye. Aβ phase 3 shows increased tracer retention compared to controls that can be measured by assessing SUVRs [7, 56]. Below the PET images SUVR thresholds are provided to distinguish Aβ phases 3, 4, and 5 from controls and from one another. The SUVRs were determined with pons as reference region [56]. Images in **b** are reproduced from Thal et al. 2015 [7] under CC BY-NC-ND license and with permission. The figure was produced in the context of a GE-Healthcare clinical trial: ClinicalTrials.gov identifier NCT01165554

Multiple types of Aβ plaques exist [47–52]. All of them contribute to the hierarchical sequence that characterizes the progression of Aβ pathology in the AD brain [49]. Diffuse non-neuritic plaques represent, in this context, an early-stage of Congo-red negative accumulations of Aβ that can—depending on the brain region of occurrence—develop into neuritic plaques [47, 48, 53]. This evolution occurs when amyloid aggregates associate with dystrophic neurites and reactive glial cells [47, 48, 53]. The Consortium to Establish a Registry for AD (CERAD) score represents a semi-quantitative measure based on the density of neuritic plaques, varying from none to frequent [54]. Given that neuritic plaques represent only Aβ plaques with dystrophic neurites usually containing p-τ, the CERAD score does not measure the extent of the Aβ pathology but rather represents the co-occurrence of Aβ and p-τ in plaques. The assessment of the entire extent of Aβ plaque pathology is done by assessing the phases of Aβ plaque distribution in the brain according to Thal et al. (Aβ phases) [55]. The first spot where amyloid plaques were found varies among places in the frontal, parietal, temporal, or occipital cortex [55]. After initiation in the neocortex (Aβ phase 1), Aβ plaques develop also in allocortical regions (Aβ phase 2), basal ganglia and diencephalon (Aβ phase 3), the midbrain and the inferior olivary nucleus (Aβ phase 4), and finally in the cerebellum and the pons (Aβ phase 5) (Fig. 2). The phases of Aβ plaque pathology correlate with the Braak NFT stages and confirm disease progression over a span of 20 to 30 years [55].

The National Institute on Aging and the Alzheimer’s Association (NIA-AA) developed a scoring system that incorporates the Aβ phases, Braak NFT stages, and CERAD neuritic plaque scores into a global ABC score. In this score, A indicates the Aβ phase and ranges from A0 (no Aβ plaques) to A3 (final Aβ phases 4–5), B indicates the Braak NFT stage and ranges from B0 (no tangles) to B3 (final Braak stages V-VI) and C indicates the CERAD stage and ranges from C0 (no neuritic plaques) to C3 (frequent neuritic plaques). Based on this ABC score, the extent of AD neuropathological changes (ADNC) can be divided into “Low”, “Intermediate” and “High” categories [44] (for A- and B-score see also Figs. 1, 2). In this context, it is essential to note that the brain stem stages of p-τ pathology are not covered by the ABC score and technically fall under the category B0 = no NFT pathology.

Despite the typical hierarchical spreading patterns of Aβ and p-τ pathology, heterogeneity among the local severities of Aβ and p-τ pathology have been described [57]. Best known are the p-τ pathology distribution related subtypes of AD [58]. The “typical “ variant is characterized by a balanced severity of p-τ pathology in the cortex and the hippocampus as predicted by the Braak NFT stage. The limbic-predominant subtype shows predominant p-τ pathology in the hippocampus whereas other brain regions are less severely affected. This is in contrast with the hippocampal sparing subtype, which is characterized by less severe hippocampal involvement but severe cortical p-τ pathology [58]. In autosomal dominant AD cases, end-stage NFT and Aβ plaque pathology is usually seen at autopsy with specific plaque types such as cotton-wool plaques [59–64]. Coarse grained plaques were reported in early onset AD [65]. In patients with Down syndrome, amyloid plaque pathology starts to develop as early as 12 years of age [66]. Later, the pathology shows all features of AD even cotton wool plaques [67]. Only the inflammatory reaction in Down syndrome shows a spectrum of involved microglial cell types that differs from classical sporadic AD [68]. The development of Aβ plaques is frequently associated with that of CAA, which is present in 80% to 100% of symptomatic AD cases [69–72].

Aβ and p-τ are not restricted to the brain. They have also been reported in the retina [73–80]. p-τ pathology frequently occurs in the retina and follows a distinct sequence in which the layers of the retina become involved: stage 1 = affection of the outer plexiform layer; stage 2 = additional affection of the inner nuclear layer; stage 3 = additional affection of the inner plexiform layer [78]. Current studies see retinal p-τ pathology in AD but also in non-AD cases. This led to the idea that a primary retinal tauopathy (PReT) could represent a precursor lesion for retinal AD manifestation [78]. Interestingly, retinal p-τ pathology was also associated with a worsening of vision [78]. Experiments in τ-transgenic mice showed that the occurrence of p-τ pathology in retinal ganglion cells is associated with a reduced ganglion cell density [81]. In contrast, retinal amyloid pathology is less frequently observed and often represents very small lesions [74, 76, 78, 82]. Often, visualization of Aβ pathology in the retina requires specific staining techniques (flatmount technology that stains the retina or pieces of the retina as one flat mounted piece) [74]. The prevalence of retinal Aβ varies among different studies but is mainly observed in symptomatic AD cases in neuropathological studies [73, 74, 76, 78, 82]. Moreover, CAA-affected blood vessels in the retina were also reported [75].

## Neuropathology of co-pathologies in AD brains

In addition to the AD hallmark lesions, other pathologies like vascular lesions, transactive-response DNA-binding protein 43 (TDP-43), αSyn pathology [19–23, 25–30], and granulovacuolar degeneration (GVD) [83–85] frequently occur in the elderly brain. Moreover, neuroinflammatory response to AD pathology impacts disease progression [86–90].

Vascular lesions, such as bleedings and infarcts are often found in the elderly brain [28, 91–94] and can have various causes. For example, atherosclerosis with thrombosis, thromboembolic occlusion of blood vessels and cardiogenic thromboembolic events are AD independent causes of brain infarcts [95–98]. Similarly, hypertensive arteriopathy is an AD-independent cause of intracerebral hemorrhage [95, 98, 99]. CAA, on the other hand, can also cause cerebral infarcts and bleedings [28, 70, 100, 101]. Given that CAA is the result of the accumulation of the same Aβ peptide in the vessel wall that also accumulates in amyloid plaques and given the strong association of CAA with AD-related β-amyloidosis, there is a potential link between CAA-related vascular lesions and AD [1, 35]. This is further supported by the fact that mouse models overexpressing transgenic amyloid precursor protein (*APP)* via a neuron-specific promoter produce not only Aβ plaques but also vascular Aβ deposits, i.e., CAA [102]. Moreover, CAA allows the distinction of two types of CAA which are associated with pathogenetically different forms of AD [103, 104]: Capillary CAA (CAA type 1) is strongly associated with the apolipoprotein E (*APOE*) ε4 allele whereas CAA cases lacking capillary amyloid deposits (CAA type 2) is not [105]. Moreover, CAA type 1 is associated with blood flow disturbances as shown in an animal model [106], and with the manifestation of allocortical microinfarcts in the human brain [107].

TDP-43 pathology in neuronal cytoplasmic inclusions and threads in elderly individuals with or without signs of amnestic dementia has recently been termed “limbic predominant age-related TDP-43 encephalopathy neuropathological changes “ (LATE-NC) [108]. LATE-NC occurs by definition either on its own or together with ADNC. Interestingly, TDP-43 aggregates are present in 20% to 74% of AD cases in which they can interact with p-τ in NFTs [21, 24, 109–114]. LATE-NC starts in the amygdala, hippocampus and entorhinal cortex (stage 1). From there it increases in severity, expands into the perirhinal cortex (stage 2) and later into the frontal cortex (stage 3) [20, 115]. With increasing age, the prevalence and stage of LATE-NC increases [22, 23]. TDP-43 pathology can also be seen in the retina. However, retinal TDP-43 pathology observed so far was either related to amyotrophic lateral sclerosis (ALS) or frontotemporal lobar degeneration (FTLD)-TDP [116–118]. About 43% to 63% of AD cases exhibit αSyn Lewy body pathology [119–122]. This pathology is characterized by Lewy bodies in neurons and Lewy neurites consisting of αSyn aggregates and is seen in AD cases with or without Parkinson motor symptoms. αSyn pathology in AD patients influences the clinical presentation (e.g., higher frequency of memory symptoms compared to pure dementia with Lewy bodies (DLB) and higher frequency of visual hallucinations, REM sleep behavior disorder or autonomic dysfunction compared to pure AD) and increases the rate of cognitive decline and mortality compared to pure AD or DLB [123–126]. The spread of αSyn pathology has been described to follow either a classical caudo-rostral distribution pattern [127] or alternatively the amygdala-predominant pattern [128–132]. The caudo-rostral distribution pattern starts with Lewy bodies in the dorsal nucleus of the vagal nerve (stage 1), extending into the locus coeruleus (stage 2), substantia nigra (stage 3), basal nucleus of Meynert and amygdala (stage 4), hippocampus (stage 5), and, finally, to the neocortex (stage 6) [127, 129, 133, 134]. In cases with the amygdala-predominant pattern, the amygdala is often affected without or with very mild accompanying involvement of the brain stem nuclei [128–130, 135, 136]. Interestingly, amygdala-predominant αSyn pathology has been frequently reported in AD cases [129, 136, 137]. At the cellular level, pathological αSyn accumulation has been reported in these cases within the same neurons that also bear NFTs [114, Ishizawa, 2003 #525, 129]. The prevalence of αSyn pathology in the general population is lower than that of AD hallmark lesions or LATE-NC [23]. Moreover, in the oldest old, the frequency of αSyn pathology decreases likely due to a lower life expectancy of cases with αSyn pathology compared to those without [23]. αSyn pathology has also been described in the retina of Parkinson’s disease patients and of patients with DLB [138–140].

GVD is neuropathologically characterized by large cytoplasmic vacuoles in neurons containing dense granules and a high number of proteins, which are often phosphorylated, including p-τ and pTDP-43 but also casein kinase 1δ/ε, charged multivesicular body protein 2B, and lysosome-associated membrane protein 1 occurring in the aging and demented brain [83, 141–143]. The latter proteins suggest that lysosomal degradation and autophagy may play a role in the development of GVD [143, 144]. GVD also contains the activated components of the necrosome, i.e., the executor complex for necroptosis, which is a distinct form of regulated cell death [145, 146]. The development of GVD starts in the CA1/subiculum region of the hippocampus, followed by expansion into the entorhinal and temporal cortex, into the amygdala and finally into the frontal cortex [85]. GVD has been reported in multiple neurodegenerative disorders including AD, ALS, Guam disease, etc. [85, 142, 143, 147]. In this context, the most advanced stages of GVD expansion were restricted to AD cases [85]. Interestingly, the GVD frequency correlated negatively with the neuronal density in the CA1 region of the hippocampus as well as in layer III of the frontal neocortex in AD cases [145]. Currently, no attempts have been made to detect GVD in vivo with biomarkers.

Finally, neuroinflammation is frequently found in AD cases and is associated with the disease development [48, 87, 90, 148–151]. Interestingly, late-stage AD cases showed a reduction of activated HLA-DR-positive microglial cells often exhibiting a conversion into the phagocytic phenotype [151]. With attention to single cell transcriptomic profiles, distinct types of microglial cells have been identified with a specific type of disease-associated microglial cells (DAMs) [152–154]. The inflammasome pathway had been identified as an important pathway in the context of AD [155–157]. Moreover, inflammasome activation is part of the pyroptosis pathway [158]. Pyroptosis is an inflammatory type of regulated necrosis and can cause cell death in multiple ways [158, 159]. In AD, the pyroptosis pathway is activated in microglial cells, astrocytes, and neurons. However, in each of the cell types, this activation is triggered via a different pathway [160]. Moreover, neuronal and microglial pyroptosis pathway activation both contribute independently to neuron death in AD [160, 161].

## Sequence of neuropathological events throughout the pathogenesis of AD and its modification by co-pathologies (Fig. 3)

**Fig. 3:**
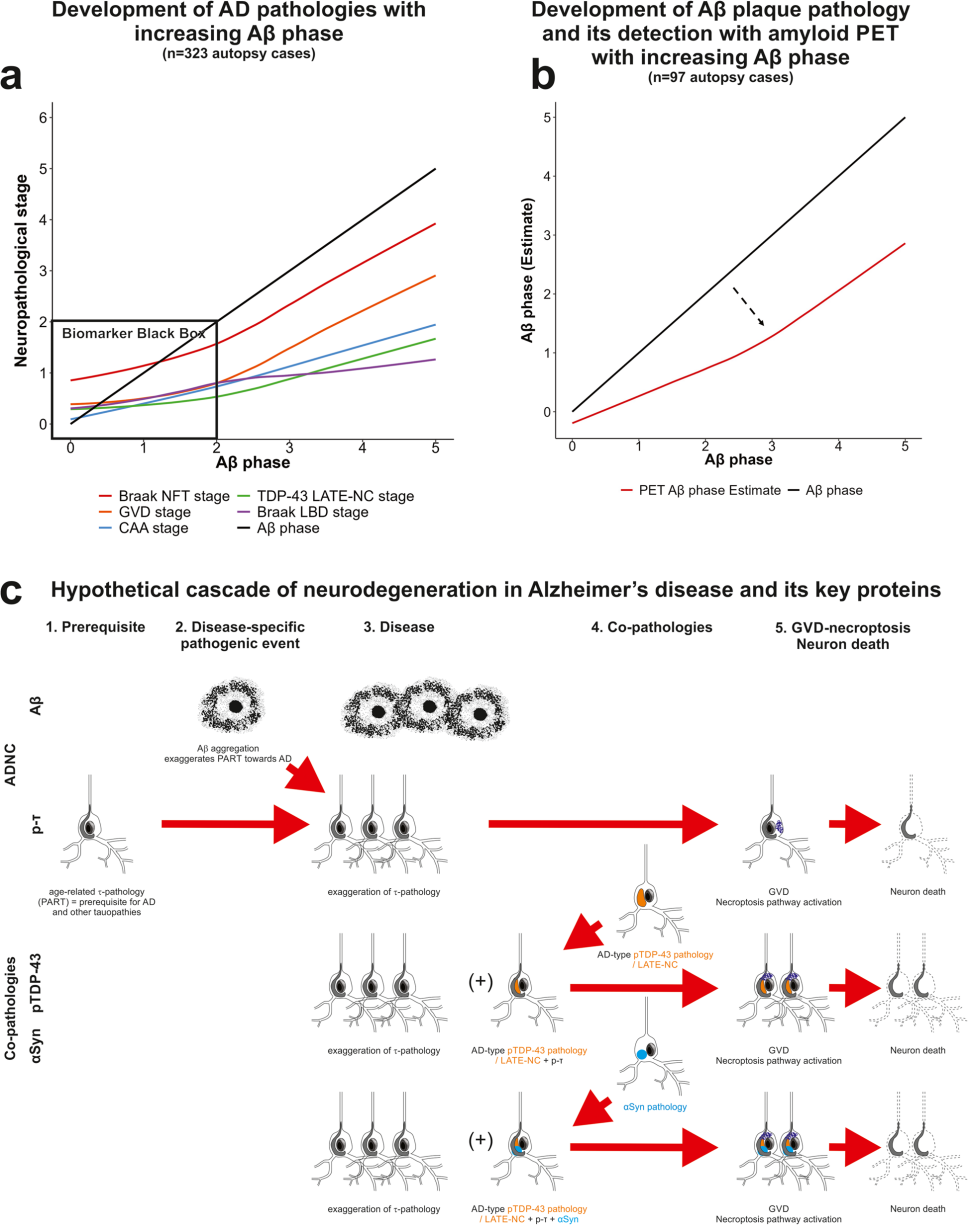
a Development of Aβ phases (0–5), Braak NFT stages (0-VI), GVD stages (indicative for necroptosis pathway-related neurodegeneration; 0–5), CAA stages (0–3), LATE-NC stages (representing TDP-43 pathology; 0–3), and Braak LBD stages (0–6) during the course of AD as represented by the Aβ phase in 323 autopsy brains using a locally weighted polynomial regression method based on the average pathology stages at each respective Aβ phase. The trajectory of each pathology is depicted by a line diagram with Aβ phase as x-axis. Note that not all AD cases exhibited LBD pathology or LATE-NC and that the resulting graphs include both cases with and without the respective pathologies. b To illustrate the sensitivity of ^18^F-flutemetamol amyloid PET compared to the neuropathological Aβ phases, we show a line diagram obtained through locally weighted polynomial regression based on the average Aβ PET stage at each respective Aβ phase. The Aβ phase is depicted by the x-axis and the y-axis shows the estimates for the Aβ phases dertermined with a given method. The black line represents the neuropathological Aβ phases whereas the red line represents the PET Aβ phase estimates according to Thal et al. [56] as measurement for ^18^F-flutemetamol tracer retention. The black diagonal line represents the neuropathological Aβ phase and was included as reference. Amyloid PET provides reliable detection of Aβ phase 3 and higher. The arrow in b indicates when amyloid PET detects the pathology. This figure is based on previously published results covering the proper box-plot diagrams of the illustration in b [56]. The statistically relevant information for a and b is depicted in Supplementary Fig. 1. c Schematic representation of a hypothetical cascade of neurodegeneration in AD and its key proteins Aβ, p-τ, and the co-pathology-related proteins pTDP-43 and αSyn. The first step in this hypothetical cascade is the development of p-τ as PART, which becomes further accelerated by Aβ (step 2) and can lead to the induction of GVD-necroptosis and neuron death as final steps in the neurodegenerative process. In this context, it is essential to note that necroptosis is only one of the cell death pathways that can execute neuron death in AD [161]. As a third step in the neurodegenerative process, co-pathologies can exacerbate p-τ pathology and neurodegeneration as shown for pTDP-43 and αSyn [19, 25, 136, 162]. At the moment p-τ and Aβ pathology can be detected with validated biomarkers whereas reliable biomarkers for the co-pathologies are not yet available

When analyzing the relationships among the pathological parameters in AD and the co-pathologies, it became apparent that p-τ pathology, as indicated by the Braak NFT-stages, usually precedes the appearance of Aβ plaques, CAA, GVD, LATE-NC and αSyn pathology (Fig. 3) [38, 163–166]. As AD progresses, Aβ pathology takes over the lead and p-τ pathology follows (for the data shown in Supplementary Fig. 1: Sign test, *p* = 0.004 (adjusted for multiple testing); *n* = 323). In a third step, GVD starts to occur and progresses through distinct stages of distribution (for the data shown in Supplementary Fig. 1: Sign test, *p* < 0.001 (adjusted for multiple testing); *n* = 323) followed by CAA stage, LATE-NC stage and Braak LBD stage (for the data shown in Supplementary Fig. 1: Sign test, *p* = 0.001 (adjusted for multiple testing); *n* = 323), which all three rise simultaneously (for the data shown in Supplementary Fig. 1: Sign test, *p* = 1.0 (adjusted for multiple testing); *n* = 323) (Fig. 3a). Results from studying the accelerating effect of Aβ on p-τ in animal models support the hypothesis that Aβ accelerates the propagation of p-τ pathology [167–170] possibly via an interaction of both proteins with the cellular prion protein [169, 171]. Downstream to Aβ and p-τ pathology, GVD develops (Fig. 3a, c) [172–174] containing components of the activated necrosome [145] and executing the regulated cell death pathway of necroptosis [158], which leads to neuron death [145, 175]. Pharmacological inhibition of the necroptosis pathway stopped the development of GVD and neuron loss in APPxτ-transgenic mice [175]. Interestingly, co-pathologies such as LATE-NC, αSyn pathology, and CAA most frequently follow the neurodegenerative process parallel to necroptosis pathway activation indicated by GVD (Fig. 3a, c). By doing so, co-pathologies are capable of accelerating the degenerative process as shown for pTDP-43 in LATE-NC, which interacts with p-τ [109] and is associated with increased p-τ seeding and propagation activity [176]. Additionally, accelerated neuron loss has been associated with an increase of GVD with necroptosis pathway activation [162]. These synergy effects were functionally demonstrated in model systems [177, 178]. Very recently, hippocampal and amygdala neurons were found to contain co-localized p-τ, pTDP-43 and αSyn [136] indicating that protein interactions in AD are not limited to bilateral interactions but can also be multilateral. Structural equation modelling indicated that also αSyn pathology and pyroptosis contribute to the severity of neuron loss in AD [136, 161].

To conclude, the pathological hallmark lesions in AD interact, at least indirectly, with one another. Aβ is, in this context, an accelerator for p-τ pathology. p-τ is capable of triggering neuron death by inducing GVD-linked necroptosis. Pyroptosis activation, TDP-43 and αSyn pathology presumably have additional effects on driving neurons into cell death.

In parallel, CAA, especially CAA type 1, also impacts neuronal survival as this type of CAA leads to the development of blood flow disturbances and microinfarcts [106, 107]. Other authors also reported an association of severe CAA with cerebral microinfarcts [101]. Accordingly, CAA can contribute to the degenerative process in AD via additional blood flow alterations.

## Biomarkers in the clinics—relationship with neuropathology

**Fig. 4:**
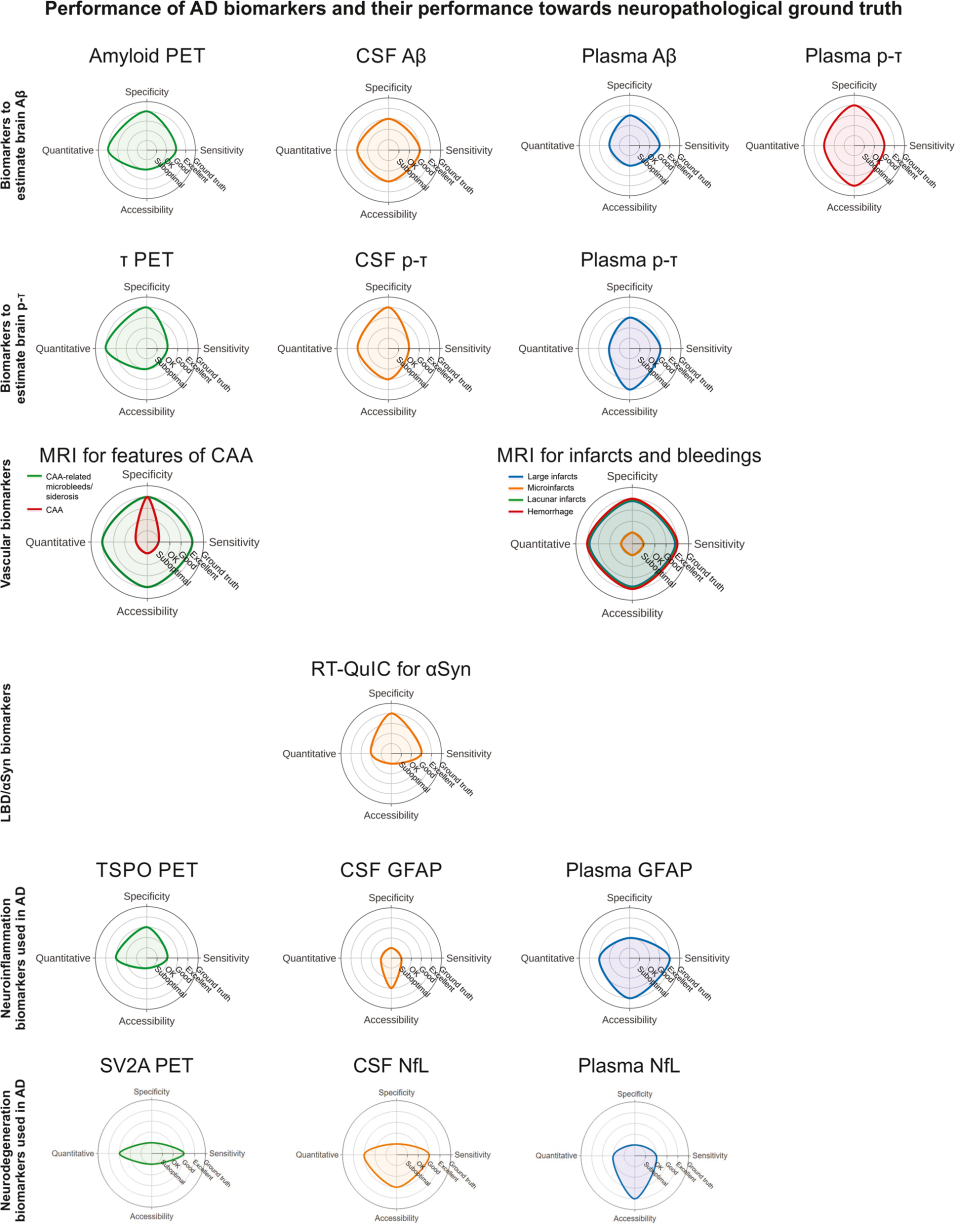
Estimation of biomarker performance (sensitivity, specificity, quantitative accuracy, and accessibility) targeting the estimation of the underlying neuropathology as ground truth. Imaging, CSF and blood biomarkers are compared for the detection of Aβ pathology, p-τ pathology, vascular pathology (CAA, infarcts and bleedings), αSyn pathology, neuroinflammation and neurodegeneration markers. The estimation was made in accordance with the literature cited in this article. Radar plots were generated using Plotly. Notes: Plasma p-τ is more specific for estimating Aβ pathology than p-τ pathology [15, 186]. The detection of CAA-related microbleeds is very specific and sensitive for symptomatic CAA cases. The bulk of the CAA cases without bleedings and siderosis that can be detected at autopsy could not be detected with MRI [187, 188]. Infarcts and hemorrhages can be excellently diagnosed by MRI. Only microinfarcts usually escape due to resolution restrictions in MRI imaging [179]. As neuroinflammation markers we included those markers that are used to determine AD-related neuroinflammation. These are the TSPO PET and measurements of glial fibrillary acidic protein (GFAP) in the CSF and blood plasma. Note that ground truth as the highest quality level does not apply to the accessibility parameter. For the determination of the degree of neurodegeneration we focus on CSF and plasma NfL as the best established fluid marker for this purpose and SV2A PET as synaptic marker to document synapse loss. MRI for determining the level of atrophy and total CSF τ are further markers for neurodegeneration but were not included in this figure as in our opinion NfL is superior to total CSF τ and synaptic PET provides a better view on the morphological correlate of neurodegeneration, i.e., a reduction of synaptic density even before substantial atrophy becomes visible

The AD hallmark pathologies Aβ plaques and NFTs, as well as certain co-pathologies, can be estimated ante-mortem using specific biomarkers. An overview about the underlying pathologies and their related biomarkers is provided in Table 1. Specificity and sensitivity towards neuropathological ground truth has been estimated according to the current literature and these estimates are depicted in Fig. 4. In the following paragraphs we introduce the respective PET and fluid biomarkers, first for biomarkers of AD hallmark pathologies, second for disease progression, and third for frequent co-pathologies, and discuss their diagnostic potential. Although included in Fig. 4 for completeness, MRI for vascular lesions and CAA as well as αSyn and translocator protein (TSPO) PET will not be discussed in detail. We refer to the respective literature [179–185].

**Table 1:** AD biomarkers, their neuropathological correlates, and diagnostic and prognostic/monitoring potentials

Biomarker	Neuropathological correlate	Diagnostic potential		Prognostic/monitoring potential	
		1st line	2nd line	1st line	2nd line
MRI	Infarcts	X			
	Hemorrhage	X			
	Microbleeds/CAA	X			
Amyloid PET	Amyloid plaques	X			
τ PET	NFTs	X		X	
TSPO PET	Neuroinflammation			tbd	
SV2A PET	Synapse density			tbd	
αSyn PET	Lewy bodies/MSA	tbd			
CSF Aβ_1–42_	Amyloid plaques	X			
CSF Aβ_42/Aβ40_ ratio	Amyloid plaques	X			
Total τ	Neurodegeneration				X
CSF p-τ^181^, p-τ^217^, p-τ^231^	NFTS	X		X	
CSF RT-QUIC	Lewy bodies/ MSA	X			
CSF GFAP	Neuroinflammation			X	
CSF NfL	Neurodegeneration			X	
Plasma Aβ_42/Aβ40_ ratio	Amyloid plaques		X		
Plasma p-τ^181^, p-τ^217^, p-τ^231^, p-τ^205^	NFTs	X (p-τ^217^)		X	
Plasma p-τ181, p-τ^217^	Amyloid plaques		X		
Plasma RT-QUIC	Lewy bodies/MSA		tbd		
Plasma EV-pTDP-43	pTDP-43 inclusions		tbd		tbd

*tbd* To be determined

Racial disparity in the levels of some biomarkers have been described [189, 190] but comparison of neuropathology with imaging or fluid biomarkers in end-of-life studies is, today, mainly restricted to US or European cohorts [8, 9, 11, 191, 192]. Further studies comparing biomarker levels in individuals of Caucasian, African, Asian, American, and Australian origin to pathological ground truth in end-of-life studies are required to clarify whether racial disparity has influence on the quality of biomarker estimates. Given the lack of these data, we will focus on the results obtained mainly in Caucasian cohorts. This is a limitation of this review.

## Biomarkers of AD hallmark pathologies

### Amyloid PET

PET imaging is an opportunity to visualize specific pathological processes using radiolabeled ligands administered intravenously that bind specifically to their target. In the case of amyloid PET, Aβ aggregates in the brain represent the target. Pittsburgh compound B (PiB) was the first developed amyloid tracer but includes ^11^C as radioactive isotope whose short half-life (20 min) limits its use in clinical settings [193, 194]. Today, three amyloid tracers incorporating the ^18^F isotope (110 min half-life), namely ^18^F-florbetapir [9], ^18^F-flutemetamol [195], and ^18^F-florbetaben [8] are commercially available and have been approved for clinical use after undergoing phase 3 end-of-life trials [8, 9, 191]. Regulatory approval of amyloid PET imaging tracers is based on standardized procedures for visual interpretation of the scans by trained readers. These procedures result in positive or negative visual reads. All aforementioned amyloid tracers specifically label amyloid aggregates in the brain but their sensitivity only allows the detection of moderate to severe amyloid pathology whereas early phases (amyloid phases 1 and 2) of β amyloidosis could not be distinguished from age-matched control brains. These data have first been obtained for ^18^F-flutemetamol and ^11^C-PiB tracers [7, 11, 56] and later for all four tracers in a study in which standardized uptake value ratios (SUVRs) were converted to Centiloids allowing direct comparison of tracer measurements [196]. Definition of amyloid tracer retention tracer thresholds (in SUVRs or Centiloids) allow principally a distinction between the different Aβ phases from phases 0–2 vs. 3 to 5 [56, 196, 197]. Longitudinal follow-up studies comparing SUVR measurements or Centiloid values with the referring post-mortem Aβ phases determined in end-of-life studies, revealed that Aβ tracer retention increases in parallel with Aβ deposition in the brain, starting from Aβ phase 3 [56, 198–202]. In addition, to the neuropathological hierarchical pattern of Aβ plaque deposition, amyloid PET allowed the identification of several cortical areas that are more prominently affected than others [203]. Variation of the primarily affected neocortical areas among groups of cases was reported [204]. Today, the approved indication for amyloid PET is the evaluation of neuritic amyloid plaque density in individuals with cognitive impairment who are being evaluated for Alzheimer’s disease or other causes of cognitive decline [205]. For clinical trials, amyloid PET has played a critical role in confirmation of amyloid positivity for amyloid lowering drug trials and for evaluating the therapeutic effect of these drugs. Amyloid PET has also been instrumental in the definition of the asymptomatic stage of AD.

### τ PET

Currently, the most widely used ^18^F-labeled τ PET ligands [206] are ^18^F-flortaucipir,^18^F-MK6240, ^18^F-PI2620, ^18^F-RO948, and ^18^F-GTP1. Other τ PET tracers have been abandoned based on company strategic decisions, such as ^18^F-JNJ067 [207], or because of lack of specificity for p-τ, such as ^18^F-THK5351 and ^18^F-THK5317. τ PET ligands are sometimes grouped as first (such as ^18^F-flortaucipir) versus second-generation tracers (such as ^18^F-MK6240, ^18^F-PI2620, ^18^F-RO948, and ^18^F-GTP1) [206]. The second-generation tracers show no off-target binding near the medial temporal cortex (choroid plexus) and less off-target binding in the striatum [208] (Fig. 1b). According to the end-of-life diagnostic phase 3 trial of ^18^F-flortaucipir [209], a binary visual read of ^18^F-flortaucipir PET as either “not consistent with AD” or “consistent with AD” can detect neuropathological Braak V-VI with high sensitivity and average-to-high specificity, meeting the primary endpoint. In this study, a positive visual read, i.e., the visual analysis indicated “consistent with AD”, could only be made if the tracer uptake extended beyond the medial and anterior lateral temporal cortex because nonspecific binding of the ligand to the choroid plexus renders evaluation of medial temporal binding of the ^18^F-flortaucipir tracer challenging. Around 75–80% of cases in Braak stage III or IV were read as negative. This pivotal study contributed to the Food and Drug Administration (FDA) approval of ^18^F-flortaucipir as a radioactive diagnostic agent for adult patients with cognitive impairment being investigated for AD. The ability of ^18^F-flortaucipir to detect neuropathological stage Braak IV or higher was confirmed in a second, academic study [210]. The degree of in vivo neocortical ^18^F-flortaucipir signal correlates closely with post-mortem p-τ load from anti-p-τ^S202/T205^ immunostaining [10, 210], and less so in limbic regions [10]. τ PET positivity typically starts near symptom onset. Similar to ^18^F-fluorodeoxyglucose (FDG) PET, but in contrast to amyloid PET, the topographical extent of τ PET positivity holds prognostic value: more widespread τ PET positivity is associated with faster cognitive decline [211]. τ PET, therefore, contributes to biological staging of the disease [212].

Results obtained with first-generation τ PET tracers may not be generalizable to the second-generation tracers because of differences in signal amplitude, off-target binding and meningeal uptake. Based on a direct within-subjects comparison, accuracy based on visual reads, was similar between ^18^F-flortaucipir and ^18^F-MK6240 [208]. ^18^F-MK6240 retention had a dynamic range that was 1.7 to 1.9 times larger than that of ^18^F-flortaucipir. A higher dynamic range is advantageous for all research based on semi-quantitative assessments, such as longitudinal within-subject comparisons or correlational analyses. Given the absence of choroid plexus retention and the higher dynamic range, it is possible that the second-generation τ PET tracers may detect earlier stages of p-τ aggregation than ^18^F-flortaucipir but that remains to be demonstrated.

According to an in vivo ^18^F-flortaucipir τ PET study, the ‘classical’ distribution defined neuropathologically by Braak and Braak [43] was present in AD cases as depicted in Fig. 1 [45, 213] but only in 30%, while other cases showed medial temporal sparing on τ PET (20%), a posterior occipitotemporal pattern (30%) or a left-lateralized temporoparietal phenotype (20%) [214]. The prevalence of these patterns will probably strongly depend on the type of cohort, e.g. research and academic memory clinic based, and likely do not reflect the population-based prevalence. The topographical heterogeneity fits with the concept of different neuropathological patterns of p-τ distribution, limbic-predominant (more frequent in older cases) or hippocampal sparing [58]. The ability of τ PET to monitor the topographical pattern of increased τ aggregates renders them very attractive as surrogate markers for assessing the effect of study drugs targeting τ aggregation in AD.

In AD, p-τ occurs under different forms, as explained above. Furthermore, different stages of NFT maturity have been described based on staining affinities [37, 215]. The precise details of which AD-related p-τ aggregates are mostly responsible for τ PET positivity (neuropil threads, different types of NFT, maybe also τ astrogliopathy) is still unresolved. However, in vitro autoradiography binding assays proved the specific binding of the τ-PET tracers showing a similar distribution as the immunohistochemical staining with antibodies against p-τ although varying in sensitivity and specificity [216–222].

Neuropathologically, primary age-related tauopathy (PART) refers to the presence of NFTs in the absence of amyloid plaques. In a large study of more than 1,840 cases scanned with ^18^F-MK6240 [223], among the amyloid PET negative healthy controls, 8% showed increased τ PET signal in medial temporal cortex, while the remainder was τ PET negative. The opposite pattern, amyloid positivity without τ PET positivity is much more common and is almost the default pattern in the asymptomatic stage of AD. Among amyloid PET negative cases with suspected non-AD pathophysiology (SNAP) [224], 13% had increased medial temporal τ PET signal and 25% had increased medial temporal and neocortical τ. Clearly, amyloid PET negativity does not preclude τ positivity although the most typical sequence is that amyloid PET positivity precedes τ PET positivity. Possibly, PART accounts for these relatively rare cases of medial temporal τ PET signal in the absence of amyloid positivity, but a post-mortem study in patients who had received an ^18^F-flortaucipir study showed no tracer retention in PART [210]. Alternatively, the amyloid PET may be false negative.

### Core CSF biomarkers for ADNC diagnosis

Given the relatively high costs for PET imaging and the limited access to this technology, cheaper and easier accessible biomarkers were needed. As body fluids can be taken easily and sent to laboratories that offer proper expertise, these fluid biomarkers are easier to access, and analysis is cheaper. Today, CSF biomarkers are well established whereas blood biomarkers are currently emerging and may replace the CSF biomarkers in the near future. CSF core AD biomarkers reflecting amyloid plaques and τ NFTs have been incorporated in the diagnostic criteria for supporting in vivo AD diagnosis since 2011, and include CSF Aβ_1–42_, total τ and phosphorylated τ at threonine181 (p-τ^181^) [212, 225].

CSF Aβ_1–42_ levels are typically lower in AD patients than in healthy elderly or patients with other dementia types (e.g. FTLD) as a result of sequestration to the brain, where it aggregates [226, 227]. However, low CSF Aβ_1–42_ levels have relatively low specificity as they can also be decreased in CAA, hydrocephalus, cerebral microangiopathy, Lewy body dementia and other neurological disorders (Fig. 4) [228–235]. Since CSF Aβ_1–40_ levels remain unchanged in AD, specificity can be substantially improved by using the Aβ_1–42_/Aβ_1–40_ ratio as it compensates for interindividual differences in total Aβ production [236–239]. In addition to Aβ, τ levels in CSF also serve as core diagnostic biomarkers for AD. In contrast to Aβ, τ is an intracellular protein that is thought to be released in the CSF through neurodegeneration, a process not specific to AD. CSF levels of p-τ^181^, on the other hand, are specifically increased upon the presence of AD pathology and are typically not altered in other neurodegenerative disorders [240]. Neuropathology as well as PET-based studies have shown that CSF-based changes in total τ as well as p-τ^181^ (e.g. at Aβ phase 4 or 5) follow both CSF-based Aβ_1–42_/Aβ_1–40_ changes and subsequent PET-based Aβ changes [237, 241–244]. Pathological levels of CSF total τ or p-τ^181^ may therefore not necessarily be present in all symptomatic cases with amyloid biomarker proven AD in a prodromal or early dementia phase (lack of sensitivity; Fig. 4) [212, 225].

### Translation of core CSF AD biomarkers to blood – relationship to neuropathology

Although CSF- and PET-based methods have transformed the in vivo diagnosis of AD in expert centers who have access to these methods, their invasive nature, relatively high cost in the case of PET, and dependence on specialized facilities make them impractical for large scale testing. Blood-biomarkers provide a more accessible and scalable alternative, which may transform the diagnostic work-up of cognitive disorders at the population level. However, blood-based biomarker development has faced its own challenges. The up to 100 times lower levels in blood than in CSF, the contribution of peripheral biomarker production to blood, which is already a very complex matrix [245], as well as the influence of proteolytic degradation [246] and co-morbidities on blood-based biomarker levels all complicate the determination of their relationship with brain-level changes [247–249]. Nevertheless, technological advancements in the immunoassay field addressed the limitations of traditional first-generation enzyme-linked immunosorbent assays (ELISAs) typically used for biomarker quantification in CSF. These advancements include the development of ultrasensitive assays, which operate on the same principle as traditional ELISAs, where an antigen is recognized by an immunocomplex, and upon addition of a substrate, generates a detectable and quantifiable signal. However, these ultrasensitive methods reach higher sensitivity using advanced detection techniques such as (electro)chemiluminescence (Mesoscale Discovery (MSD), Lumipulse and Elecsys platforms) or bead-based single molecule digital detection (single molecule array (Simoa) platform) rather than relying on colorimetric signals. Additionally, improvements in ELISA design as well as increased attention for preanalytical parameters (e.g. centrifugation delay and parameters, sample tube, storage delay) have allowed quantification of AD-related proteins in blood.

### *Plasma* Aβ_1–42_/Aβ_1–40_*ratio*

Similar to CSF, the plasma Aβ_1–42_/Aβ_1–40_ ratio is decreased in individuals demonstrating ADNC at autopsy, regardless of clinical symptoms [192, 250, 251]. Several studies reported a decreased plasma Aβ_1–42_/Aβ_1–40_ ratio at early disease stages, i.e., intermediate ADNC and moderate plaque burden as determined by CERAD staging, after which it plateaued [192, 250]. This is comparable to the temporal trajectory observed for CSF Aβ_1–42_/Aβ_1–40_ [236, 252]. However, whereas the CSF Aβ_1–42_/Aβ_1–40_ ratio is reduced by more than 50% in AD [233], plasma-based reductions are less pronounced (10–20%) [253–255]. Importantly, the performance of plasma Aβ_1–42_/Aβ_1–40_ to detect AD is highly dependent on the assay used for its quantification. Whereas plasma Aβ measurements by first-generation Aβ ELISAs lacked performance as AD biomarkers, novel ELISAs that incorporate N-terminal – rather than midregion – antibodies, employ improved conjugation methods, and demonstrate comparable performance to equivalent ultrasensitive Simoa assays [253, 254]. Yet, highest performance of the Aβ_1–42_/Aβ_1–40_ ratio as an AD biomarker is obtained when it is quantified by mass-spectrometry based methods rather than immunoassays [253]. For example, classification accuracy for detecting amyloid status across the AD continuum – as determined by areas under the curve (AUC) in receiver operating characteristic (ROC) analyses—ranges between 0.83 and 0.97 for mass-spectrometry methods [253, 255–257] and between 0.62 and 0.79 for immunoassays (e.g., Simoa, ELISA) [253, 254, 258–261].

### Plasma total and p-τ

Not all core CSF biomarkers have been successfully translated to blood. Blood-based total τ levels, for instance, are lower than those of Aβ_1–42_ and Aβ_1–40_, necessitating ultrasensitive methods for detection. However, plasma total τ measurements using either Simoa or Elecsys platforms have demonstrated poor correlation with CSF-based total τ and largely overlap between several neurodegenerative disorders as well as between clinical AD stages [262–267]. As a result, blood-based total τ has poor diagnostic and prognostic performance in AD [262–268]. In contrast, p-τ^181^, whose blood levels are even lower than those of total τ, has demonstrated consistent and specific elevations in plasma of patients with underlying ADNC [269, 270] when measured with ultrasensitive methods. These p-τ^181^ elevations did, however, occur in later stages than Aβ_1–42_/Aβ_1–40_ decreases (i.e., at high ADNC including frequent plaque burden according CERAD and Aβ phase 4 or 5) [192, 250, 271, 272]. Consequently, plasma Aβ_1–42_/Aβ_1–40_ correlates strongly with amyloid burden in early disease stages, whereas p-τ^181^ demonstrated a stronger correlation at later amyloid stages or once cognitive symptoms emerge than at earlier disease stages [192, 251]. Moreover, while Aβ_1–42_/Aβ_1–40_ mainly correlates with amyloid load [192, 254, 259], plasma p-τ^181^ is independently associated with both cerebral amyloid and p-τ pathology [192, 273]. Of note, the τ protein has over 40 possible phosphorylation sites and alternative p-τ species have also shown promise in AD diagnosis with different associations to cerebral p-τ pathology and Aβ pathology. p-τ^231^, for example, increases in earlier stages than p-τ^181^ in plasma as well as in CSF, yet has comparable performances to plasma p-τ^181^ beyond these earliest stages [250, 274–276]. p-τ^217^, on the other hand, has consistently shown superior performance to p-τ^181^ as well as p-τ^231^ and Aβ_1–42_/Aβ_1–40_ [277–282]. In CSF and/or plasma, p-τ^111^, p-τ^153^, p-τ^208^, and p-τ^231^ appear to be more strongly associated with amyloid, while other τ phosphorylation sites are more strongly associated with p-τ pathology, e.g., p-τ^205^ or with both to a comparable extent, e.g. p-τ^181^, and p-τ^217^ [192, 283].

When comparing p-τ plasma biomarkers, it is critically important to consider not only the measured species and employed platform, but also the assays that are used on the respective platforms. While plasma p-τ^217^ has repeatedly outperformed p-τ^181^, the Simoa and MSD assays used for p-τ^181^ quantification in these comparisons are typically based on the AT270 antibody, which cross-reacts with p-τ^175^ [273]. Since p-τ^175^ has not demonstrated AD-related changes in CSF [283], this cross-reactivity likely confounds performance of p-τ^181^. Alternatively, a p-τ^181^ Simoa immunoassay incorporating the more phospho-specific antibody ADx252 has demonstrated very comparable performances to p-τ^217^ in both CSF and plasma [268] with respect to detecting amyloid burden as well as predicting longitudinal amyloid accumulation in head-to-head comparisons (∆AUC = 0.007—0.045) [186, 284, 285]. While mass-spectrometric plasma p-τ^217^ assays did demonstrate higher performance than both p-τ^217^ and p-τ^181^ immunoassays, the difference was only minimal (∆AUC = 0.061 – 1.06, *P* < 0.027) [285], and immunoassays might therefore have higher clinical utility considering its higher throughput and ease of use. Fully-automated assays, such as p-τ^217^ and/or p-τ^181^ assays on Lumipulse and Elecsys platforms, which have recently become commercially available, will presumably further facilitate clinical implementation of blood-based biomarkers.

Advancements in blood-based biomarker diagnostics have contributed to a revision of the criteria for diagnosis and staging of AD in 2024 [212]. In addition to CSF and PET based biomarkers, blood-based levels of Aβ_1–42_/Aβ_1–40_, p-τ^181^, p-τ^217^ and p-τ^231^ have also been integrated as diagnostic biomarkers. Due to its limited diagnostic potential in blood, the use of total τ as a diagnostic AD biomarker is recommended in conjunction with Aβ_1–42_ in a hybrid ratio, rather than as a standalone biomarker [212, 226, 267].

## Prognostic biomarkers

In addition to the well-established AD-specific diagnostic biomarkers, the revised criteria also incorporate prognostic biomarkers. Prognostic fluid biomarkers can be quantified in both blood and CSF [212]. In addition to τ-related AD-specific prognostic biomarkers, some reflect phenomena that are shared among several neurodegenerative disorders and are thus not specific for AD, but are predictive for future cognitive decline, pathogenic processes or dementia onset: Neuroinflammation-, neurodegeneration-related, and synaptic biomarkers. Estimates for the sensitivity, specificity, accessibility, and the potential for quantification of the most established and the most promising of these markers are depicted in Fig. 4 and included in Tab. 1. Given the information about the stage of the disease of a given patient provided by these markers, many of them may also be well suited for disease monitoring purposes under therapy.

### τ-related prognostic biomarkers

In contrast to amyloid PET, τ PET has a high predictive value for subsequent cognitive decline over the next couple of years, both in the asymptomatic and the early symptomatic stage [211, 286–288].

The microtubule-binding region of τ (MTBR-τ^243^) is a non-phosphorylated τ biomarker that can be quantified by mass spectrometry. MTBR-τ^243^ is the main component of insoluble τ aggregates and is increased in CSF of AD patients, but not other tauopathies, in which they have shown stronger associations with τ PET than p-τ species [289, 290]. Whereas p-τ species like p-τ^181^, p-τ^217^ and p-τ^231^ have shown the highest rate of change prior to τ-PET positivity, MTBR-τ^243^ increases more rapidly in τ-PET positive individuals, indicating it might provide additional value as a prognostic or staging marker in more advanced disease stages [290, 291]. Alternatively, the phosphorylated τ biomarker p-τ^205^ in CSF is also associated with τ PET in amyloid-positive individuals and increases earlier than MTBR-τ^243^, but later than the diagnostic p-τ^181^ and p-τ^217^ biomarkers and might thus also provide prognostic and staging information [290, 292]. To which extent these τ-related prognostic CSF biomarkers can be translated to blood remains to be investigated. Another novel approach is the use of antibodies specific to brain-derived τ to detect it in blood [293, 294].

### Neuroinflammation-related biomarkers

Glial fibrillary acidic protein (GFAP), a marker of astrocytes and astrogliosis, is one of the “neuroinflammation-related markers” that has limited diagnostic utility for AD but has demonstrated prognostic potential. GFAP levels in both serum and plasma predict dementia onset as well as cognitive decline and grey matter loss in asymptomatic individuals [295–298]. Although not AD-specific, GFAP has demonstrated more pronounced elevations in plasma of AD patients than in other dementia types such as frontotemporal dementia (FTD), DLB or vascular dementia [299, 300]. Pereira et al. [301] reported that plasma GFAP levels associate with amyloid-PET, but not τ PET in non-demented individuals. Neuropathology studies, however, revealed that ante-mortem blood-based GFAP levels associate with both amyloid plaques and NFTs at autopsy [272, 302] which can be expected as both Aβ plaques and NFTs are associated with activated astrocytes [303, 304]. Moreover, when plaques and NFTs are included simultaneously in the statistical model, GFAP only associates with NFTs [192]. Remarkably, unlike other biomarkers, the magnitude of these blood-based GFAP increases is higher than those in CSF and blood-based GFAP has demonstrated higher performance to detect AD [305].

Soluble triggering receptor expression on myeloid cells 2 (sTREM2), a microglial marker, and Chitinase-3-like protein 1 (YKL-40), a marker of macrophage activity, as well as chitinase-enzyme activity are increased in AD patients, from preclinical stages, when measured in CSF, but not in plasma [306–311].

Another neuroinflammation-related biomarker is interleukin 18 (IL18) which is increased in the blood of patients with AD, ALS, FTD, or small vessel disease [312–316]. To what extent IL18 can serve as a biomarker for AD as suggested or more as an unspecific marker for neurodegeneration and small vessel disease-related neuroinflammation requires further research. Specific neuroinflammation-related pathways, such as pyroptosis, are not yet in the focus of the neuroinflammatory biomarkers for AD.

In addition to fluid biomarkers, TSPO PET as well as more novel PET tracers [317] have been used to trace neuroinflammation in the brain of patients with neurodegenerative disease including AD but this falls outside of the scope of this review [183–185].

### Neurodegeneration-related biomarkers

Neurofilament light chain (NfL) is a cytoskeletal component of neurons that is essential for the growth and stability of axons and synapses [318]. Blood-based NfL shows a strong correlation with NfL in CSF [319] and is considered a general marker of neurodegeneration as its levels are elevated in a variety of neurodegenerative disorders [320, 321] considering its increased release from degenerating neurons in the form of extracellular vesicles [322]. In contrast to GFAP, the magnitude of plasma and CSF NfL elevations is not higher in AD than in other dementia types and does not associate with amyloid or p-τ pathology at autopsy [192, 234, 250–252, 269, 300, 302]. Similar to GFAP, blood-based NfL levels in asymptomatic individuals are predictive for subtle cognitive decline and onset of dementia due to AD or other causes [295, 297, 323, 324]. One important caveat is that the sensitivity of NfL for not only cognitive disorders but also numerous other neurological disorders or co-pathologies, might complicate interpretation of its prognostic and monitoring value in clinical settings where comorbidities are prevalent [325–327]. However, for the purpose of disease monitoring, reduced NfL levels may sufficiently indicate reduction of the degenerative process under therapy.

### Synaptic biomarkers

In AD, but also other neurodegenerative disorders such as DLB and progressive supranuclear palsy, synaptic loss occurs early and precedes neurodegeneration, thereby marking one of the earliest pathological changes [328, 329]. This early involvement, combined with the strong correlation between synaptic density and cognitive decline, makes synaptic proteins particularly promising candidates as prognostic or even diagnostic markers in the early stages of the disease [330, 331]. CSF levels of several synaptic proteins have shown elevations in AD patients compared to controls [332, 333] and/or non-AD neurodegenerative disorders [333–338]. Some synaptic proteins have also shown AD biomarker potential in blood [339–342]. PET tracers targeting the synaptic vesicle protein 2A (SV2A) have been used for research purposes and showed decreased binding in several neurodegenerative disorders, including AD (reviewed in Carson et al. [343]). In neuropathological studies, the SV2A PET tracer UCB-J was shown to target SV2A with high specificity, and SV2A levels showed moderate to strong associations with the levels of the synaptic marker synaptophysin across diverse brain regions [344, 345]. Altogether this supports the utility of SV2A PET as a surrogate marker for synaptic loss in neurodegenerative disorders.

## Retinal imaging

Multiple retinal imaging methods are currently explored for their potential to estimate brain AD pathology [346, 347]. Most of these methods aim to either determine degeneration of the retina as morphological parameters for degeneration of central nervous tissue, such as OCT [346–352], or focus on the detection of amyloid plaques or pathological protein accumulation in the retina by fluorescence imaging after dye application or hyperspectral imaging [353–355]. Recently, also p-τ pathology came in the focus of retinal imaging approaches [356]. With the current knowledge about the retinal manifestation of AD pathology, the detection of Aβ plaques appears to be a later event in the retina. Detection of neurodegeneration or even p-τ pathology in the retina would allow diagnosis of AD p-τ pathology at an earlier point in time. However, the first tauopathic manifestation in the retina appears to be a precursor lesion called PReT which has also been reported in young individuals without any evidence of AD. PReT has also been reported in the context of neuroinflammatory lesions in the retina as well as in cases with glaucoma [78, 79]. Given the convincing evidence of αSyn pathology and TDP-43 pathology in the retina, [116, 117, 138, 139] further research into a specific distinction between p-τ, αSyn, and TDP-43 pathologies in the retina could lead to a better diagnosis of co-pathologies and, by doing so, to a better stratification of AD allowing the development of personalized treatment strategies depending on the spectrum of co-pathologies.

## Biomarkers for co-pathologies

Most AD patients are only given a single clinical diagnosis throughout life, but at autopsy, the majority demonstrates several co-pathologies [19, 23, 120, 357]. As mentioned earlier, αSyn aggregations are often present in patients with concomitant ADNC [19, 23, 357]. As a consequence of the common occurrence of Lewy body pathology in AD patients and vice versa AD pathology in DLB cases, DLB patients often demonstrate blood-based changes in AD-typical biomarkers like Aβ_1–42_/Aβ_1–40_, p-τ^217^ and p-τ^181^ [358]. However, neuropathological as well as imaging-based studies demonstrated that these AD biomarker changes in patients with mixed pathology are related to the underlying ADNC rather than αSyn pathology [234, 240, 250]. Given the possible influence of αSyn pathology on disease progression in AD patients [136], in vivo measures of αSyn co-pathology, when available, may provide an explanation for some of the inter-individual variability in the disease course or the treatment effects in clinical trials in AD.

In vivo measures of αSyn pathology may also be extremely useful in the diagnosis of synucleinopathies (e.g., DLB, Parkinson’s disease, multiple system’s atrophy (MSA)) and for more efficient drug trials for these disorders. αSyn PET tracers have been tested in first-in-human trials and show promise although the diagnostic accuracy may differ depending on the specific disease. One study revealed a high sensitivity in MSA with lower diagnostic performance in other synucleinopathies [359]. Very recently the αSyn PET ligand (^18F^C05-05) was presented to determine a specific signal in cases with Lewy body disease, i.e., Parkinson’s disease and DLB [360]. These reports need to be confirmed and may provide impetus for a novel field of research. Additionally, αSyn seed amplification assays (SAAs; also known as αSyn RT-QuIC test) detecting misfolded αSyn aggregates in CSF [361–365] and skin biopsies [366–368] have been validated. These SAAs leverage the intrinsic self-propagating nature of misfolded αSyn for its detection. This process involves addition of C-terminal truncated αSyn monomers to a patient sample. If misfolded αSyn seeds are present, the substrate is converted to a misfolded form, promoting aggregation through β-sheet formation [369]. Mechanical disruption of these aggregates causes fragmentation, generating new seeds for further polymerization. Repeated cycles of elongation and fragmentation lead to exponential amplification of the aggregates. The fluorescent dye thioflavin T binds β-sheets, enabling detection. In a direct comparison between ante-mortem and post-mortem CSF αSyn SAA, sensitivity and specificity to detect limbic or neocortical αSyn pathology in vivo was above 90%. However, amygdala predominant Lewy body pathology, which is common in AD, was detected with a sensitivity of only 14%, probably reflecting differences in biochemical characteristics of the different Lewy body subtypes [365, 370]. As a more accessible alternative, blood-based αSyn assays have recently been developed. Thereby extracellular vesicles (EVs) have been targeted, which are membrane-derived particles that are released into biological fluids and mediate intercellular communication or clearance of toxins [371]. In plasma of Parkinson’s disease (PD) patients, misfolded αSyn has been detected in such EVs, particularly in neuron-derived EVs [372]. Alternatively, addition of an immunoprecipitation (IP) step to RT-QuIC (IP/RT-QuIC) has allowed detection of even the smallest amounts of aggregated αSyn in native blood rather than in EVs. These blood-based αSyn levels – as determined by the IP/RT-QuIC method – are higher in PD, LBD and MSA patients compared to healthy controls and/or AD patients [373].

Other common co-pathologies like TDP-43 cytoplasmic inclusions [24, 374–376] also have a synergistic effect on cognitive impairment in AD. Development of pTDP-43 pathology-specific biomarkers would allow a more complete and pathology-based approach in clinical practice (precision-medicine) as well as subject stratification in therapeutic trials. The initial first-in-human studies of TDP-43 PET are currently starting. Preliminary evidence indicates high affinity and selectivity of TDP-43 PET tracers for FTLD-TDP pathology [377]. Hippocampal volume loss on structural MRI in older adults may also be partially explained by TDP43 pathology beyond what τ PET can explain [378]. This variance in hippocampal volume that cannot be explained by τ PET load is sometimes referred to as the “volume-uptake mismatch”. However many factors contribute to hippocampal volume beyond τ or TDP-43 aggregation.

TDP-43 fluid biomarkers are also still in an early development stage. Initial studies using ELISAs developed for determining TDP-43 [379] demonstrated elevated levels of total TDP-43 and pTDP-43 in CSF of patients with TDP-43 proteinopathies, including ALS, FTD and AD [380–382]. In blood, this assay had insufficient sensitivity to detect TDP-43 in the majority of patients, yet also demonstrated elevated TDP-43 or pTDP-43 levels in FTD, AD and/or amyotrohic lateral sclerosis (ALS) patients with detectable plasma levels [113, 379, 383]. In contrast, more recent blood-based studies using commercial assays on the ELISA or Simoa platform show decreased TDP-43 and pTDP-43 level in TDP-43 proteinopathies [384–386]. These discrepancies might be attributed to differences in assay sensitivities and TDP-43 contributions from peripheral tissues. Emerging approaches, such as detection of TDP-43 related cryptic exon neoepitopes [387–389] or quantification of TDP-43 in neuron-derived EVs [390, 391] show promise for more specific and early biomarkers. Recently, a Simoa assay of TDP-43 in small EVs (sEVs) from plasma combined with mass spectrometry measures of 3R/4R τ ratio in sEVs showed high sensitivity and specificity for distinguishing between TDP-43 proteinopathy and tauopathies (FTLD-TDP-43 and ALS versus FTLD-tau and progressive supranuclear palsy) [390]. Cryptic exon- and sEV-based assays may also hold promise for detection of TDP-43 and other co-pathologies in AD but that remains to be investigated. Of note, no interaction effect of TDP-43 pathology on plasma or CSF biomarkers reflecting AD pathology has been found in patients with mixed pathologies [234, 250, 392, 393].

## The biomarker black box

As reported, the currently available biomarkers do not allow the diagnosis of very early stages of AD pathology, such as Aβ phases 1 or 2 (Fig. 3b), the non-argyrophilic stages of p-τ pathology [38] and even the Braak NFT stages I and II. This indicates a black box of cases with low AD pathology that biomarkers currently do not detect. This has important implications on pathogenetic conclusions drawn from biomarker studies. Biomarker studies showed that Aβ levels changed first, whereas p-τ levels followed [394]. Figure 3a illustrates that this fits well with the development of AD pathology outside the biomarker black box. Within the biomarker black box, however, it becomes evident that p-τ pathology prevails at very low levels, even in cases lacking Aβ pathology, confirming the interpretation of reports on the prevalence of the respective neuropathological changes [23, 38, 395]. When carefully comparing neuropathologically defined symptomatic and asymptomatic AD cases with non-AD controls from a previously published collection of cases [23], it becomes evident that especially in the earliest stages of Aβ pathology, p-τ emerges first, showing already an increase in non-AD cases without Aβ plaques (= Aβ phase 0) (Fig. 3).

## Biomarkers in a clinical context

Given the updated clinical criteria for the diagnosis of AD, the determination of biological parameters with biomarkers is recommended [212]. At least one marker covering amyloid pathology and one covering p-τ pathology are suggested to be used for determining the biological diagnosis of AD [212]. Whether imaging, CSF, or blood-based markers of amyloid and p-τ will be used to reach this goal is left to the clinician [212]. The availability as well as cost aspects will, in this context, play a role for the individual decision. Based on these guidelines, the positivity for amyloid is already sufficient to diagnose AD [212]. Accordingly, one can discuss whether p-τ will add any additional diagnostic information. However, since p-τ markers may provide added value for determining the stage of the disease, this information may aid both differential diagnosis and disease prognosis [396–398].

In the clinic, a multimodal approach may be appropriate, e.g., based on a stepwise sequence of tests. Depending on the context of use, either specificity or sensitivity could be set at 90 or 95%, leading to a dual cut-off. With a dual cut-off a variable proportion of cases will have intermediate levels that do not allow strong diagnostic conclusions. In these selected cases subsequent amyloid or τ PET scanning may be useful to reach a higher degree of certainty. From a patient perspective, the dual cut-off approach with subsequent PET imaging in intermediate cases is attractive as it avoids excessive investigations in most cases and, at the same time, significantly reduces the rate of false-positive or false-negative diagnosis that may be seen when the investigation is limited to the fluid biomarker test [378]. If PET is not an option, patients with intermediate results will need to be informed about the degree of diagnostic uncertainty and a wait-and-see approach may be appropriate. Table 1 gives an overview about the current biomarkers and their use as diagnostic and prognostic/monitoring biomarkers. While fluid biomarkers are also in use for disease monitoring in the context of clinical trials, none of these markers have currently been validated for implementation in a clinical context.

An important ongoing effort relates to the establishment of a diagnostic interpretation algorithm that provides a standardized way of interpreting biomarkers for diagnosis of AD in its symptomatic and asymptomatic phase, which is commutable with respect to the different methods used across different laboratories. The Centiloid scale for amyloid imaging [399] is a highly successful example of such harmonization. A similar initiative is ongoing with the CenTauR scale for τ PET, however, this is hampered by the much higher heterogeneity between τ PET tracers than is the case for amyloid PET [400]. For biofluid markers, the commercialization of automated platform assays with universal thresholds as part of the regulatory approval package will also significantly enhance their diagnostic value on a very short term [401]. Artificial intelligence (AI)/machine learning may also help us to identify reproducible thresholds for diagnosis or disease staging. Especially for imaging techniques these approaches were already used in the past [197, 402–405]. AI approaches are also pursued to analyze complex biomarker datasets, e.g., for proteomics of CSF or blood samples or for analyzing genetic endophenotypes for predicting neuropathological diagnosis [401]. To conclude, fluid and PET biomarkers can significantly augment diagnostic accuracy in the clinic which is essential for disclosure of diagnosis and prognosis and the development of drug and nondrug management plans.

A critical perspective upon diagnostic and prognostic use of fluid or imaging biomarkers is the participant’s or patient’s view. Paradoxically, as a diagnosis of AD relies more and more on technical investigations, shared decision making has become more and more important so that the individual is informed about the possible outcomes of the test prior to testing and the options to choose from, including whether to perform a specific biomarker test or not. Shared decision making prior to testing is also advantageous at the time of test result disclosure as it helps the individual to prepare for the possible outcomes and avoids that information is disclosed which the individual would have preferred not to know.

## Biomarkers for use in clinical trials and personalized treatment

For the use of biomarkers in clinical trials as well as for personalized treatment, it is essential to clarify the purpose of use. In clinical therapeutic trials in AD, biomarkers have been mainly used for (1) selection of the study target population and (2) for monitoring of target engagement and disease course. Given the disease heterogeneity of AD, co-pathologies as well as distinct subtypes may deserve more attention in the future as these may affect the therapeutic response. It is important to keep in mind that biomarkers do not identify individuals with initial ADNC. Since even such “subthreshold” initial ADNC are capable of inducing seeding, heterogeneity of the control group cannot be excluded. For example, lysates from brains with initial Aβ pathology (Aβ phase 1) can induce Aβ plaque seeding and propagation in mouse models as well as maturation of Aβ towards the additional accumulation of post-translationally modified forms of Aβ [406]. Likewise, Braak NFT-stage I brain lysates induced seeding in vitro [407] and after subretinal injection in the retina of τ-transgenic mice [81] whereas propagation into the brain was not observed in this study, except for AD brain lysates from symptomatic individuals [81]. The importance of subthreshold amounts of Aβ has been further demonstrated in APP knockout mice who received AD brain seed injections [408]. Although these mice did not develop Aβ deposits, when their brain lysates were injected into seeding competent APP transgenic mice, accelerated seeding of Aβ pathology was reported to be induced by persisting seeds [408].

For the identification of AD subtypes related to the regional pattern of neurodegeneration [58] τ-PET appears to be best suited as it reflects the density of tauopathic lesions in the different areas of the brain [409, 410]. However, atrophy related approaches using MRI for atrophy mapping have also been reported, often using AI-based methods for distinction [410, 411].

Given the high frequency of comorbidities in the AD brain [19, 23, 137, 357] it is likely that co-pathologies impact the disease course and treatment effects in clinical trials. Co-pathologies should, therefore, be considered in the stratification step and, during study follow-up. AD cases with αSyn-pathology could be identified by, e.g. an RT-QuIC test, although the sensitivity was not sufficient to detect LBD properly in demented individuals [363, 364]. Hence, more sensitive biomarkers for αSyn aggregates are required. A high frequency of pTDP-43 pathology (67%) in cases with the limbic-predominant type of AD has been reported [376]. For pTDP-43 pathology detection, an EV-based assay to estimate CNS TDP-43 pathology in the blood had been developed for FTLD-TDP and ALS and may also have a potential to estimate LATE-NC [390], which remains to be determined. Moreover, for αSyn and pTDP-43 biomarkers, the boundaries of the clinical black box still need to be delineated against neuropathological standards. Even for clinical practice, proper detection of co-pathologies that offer additional treatment opportunities makes sense when aiming for personalized treatment approaches. CAA is another co-pathology that becomes clinically evident by cerebral hemorrhages and microbleeds [187, 188]. In cases with at least microbleeds, CAA can be observed by MRI [180, 188, 412]. However, the largest number of AD cases with CAA co-pathology escape clinical detection as vascular Aβ deposition, which is characteristic for CAA, cannot be distinguished from amyloid plaques by amyloid-PET because the presence of plaques already causes tracer uptake [395]. The presence of an *APOE* ε4 allele is a very strong risk factor for capillary CAA (CAA type 1) and could be used to identify AD patients at risk for capillary CAA [103, 105]. Given the low sensitivity/specificity for detecting CAA in patients, better biomarkers for CAA would be important. However, 80% to 100% of the symptomatic AD patients showed CAA of any type at autopsy [69–71, 413], i.e. the biomarker-based diagnosis of symptomatic AD in combination with APOE genotypting may already allow for a guess that CAA will also be prevalent.

Biomarkers are also used in clinical trials and for personalized medicine for monitoring treated individuals. The first Aβ targeting drugs (lecanemab and donanemab) successfully lowered amyloid levels in the brain, reached the clinical outcome criteria [5, 6, 414–416], and have been approved by the FDA, and several other regulatory authorities worldwide. In addition to documenting target engagement (e.g., lowering of Aβ or p-τ), it is also necessary to document a disease modifying effect of a given drug with respect to neurodegeneration and neuroinflammation. To do so, we need biomarkers, which recapitulate the progression of amyloid and τ pathology in longitudinal studies, and others, which document the progress of the neurodegenerative process [198, 199, 250, 260, 301, 417–420]. In longitudinal studies, p-τ biomarkers documented the course of the pathological hallmarks well and especially τ PET provides information of the progressive destruction of the brain by p-τ pathology and potential sites of interaction with Aβ [198, 211, 262, 270, 276, 421–423]. The degenerative process can also be documented in a nonspecific manner, e.g., by means of synaptic biomarkers [250, 324]. The PET imaging for synapse densities with the SV2A tracer appears to be well suited to provide information about the result of the degenerative process and to what extent it continues or stops [343, 424–427]. To estimate the contribution of neuroinflammatory changes on AD progression and its modification under therapy, plasma GFAP has been shown to be helpful [297, 299, 301, 324, 420].

## Conclusions

Current biomarkers are well suited to determine Aβ and p-τ pathology in symptomatic AD cases and in a subset of non-demented individuals with ADNC whereas early stages of ADNC escape detection and represent a “biomarker black box”. Although the currently available biomarkers represent an enormous progress for the clinical diagnosis of AD, we still need to improve their sensitivity, since the degenerative process already leads to the loss of neurons in non-demented cases demonstrating ADNC amounts below the sensitivity threshold of currently established biomarkers. Moreover, we need markers allowing a reliable detection of co-pathologies as they enable us to distinguish different subtypes or subforms of AD to improve personalized treatment approaches and possibly better stratification of patients participating in clinical trials.

Given the “biomarker black box” for the earliest disease stages, pathogenetic conclusions about the disease onset require gold standard neuropathological confirmation whereas the further course of the disease is similarly reflected by biomarkers as in neuropathological studies and can be longitudinally studied with the current biomarkers. For disease monitoring under therapy, documentation of target engagement as well as disease progression with neurodegeneration and inflammation is needed. Accordingly, different biomarkers are required that reflect the dynamics of the degenerative and inflammatory process (e.g., p-τ-, GFAP-, NfL-, or synapse-related biomarkers) rather than highly sensitive markers that reached already the plateau-phase when the clinical symptoms become evident (e.g., Aβ-related biomarkers). Biomarkers that reflect novel disease targets, such as regulated cell death pathways (e.g., necroptosis), and/or the accompanied inflammatory reaction may need to be developed in the future for designing personalized AD therapy concepts.

## Supplementary Information

Supplementary Material 1

## Data Availability

Data reanalyzed for this review are provided in the supplementary material or were published elsewhere as indicated by respective citations. Further information can be requested from the corresponding author on reasonable request.
