# Supplementary material for: Alzheimer's disease neuropathology and its estimation with fluid and imaging biomarkers
Source: Mol Neurodegener. 2025 Mar 14;20:33. doi: 10.1186/s13024-025-00819-y (PMC11907863; doi:10.1186/s13024-025-00819-y)
Supplement: Supplementary file 1 — Supplementary Material 1 [file 13024_2025_819_MOESM1_ESM.docx]

**Additional File 1:**

**Supplementary material to:**

**Alzheimer’s disease neuropathology and its estimation with fluid and imaging biomarkers**

Dietmar Rudolf Thal^1,2^, Steffi De Meyer^3,4^, Koen Poesen^3,5^, Rik Vandenberghe^4,6^

^1^ Laboratory for Neuropathology, Department of Imaging and Pathology, Leuven Brain Institute, KU Leuven, Leuven, Belgium

^2^ Department of Pathology, University Hospitals Leuven, Leuven, Belgium

^3^ Laboratory for Molecular Neurobiomarker Research, Department of Neurosciences, Leuven Brain Institute, KU Leuven, Leuven, Belgium

^4^ Laboratory for Cognitive Neurology, Department of Neurosciences, Leuven Brain Institute, KU Leuven, Leuven, Belgium

^5^ Department of Laboratory Medicine, University Hospitals Leuven, Leuven, Belgium

^6^ Department of Neurology, University Hospitals Leuven, Leuven, Belgium

**Supplementary Figure 1:**


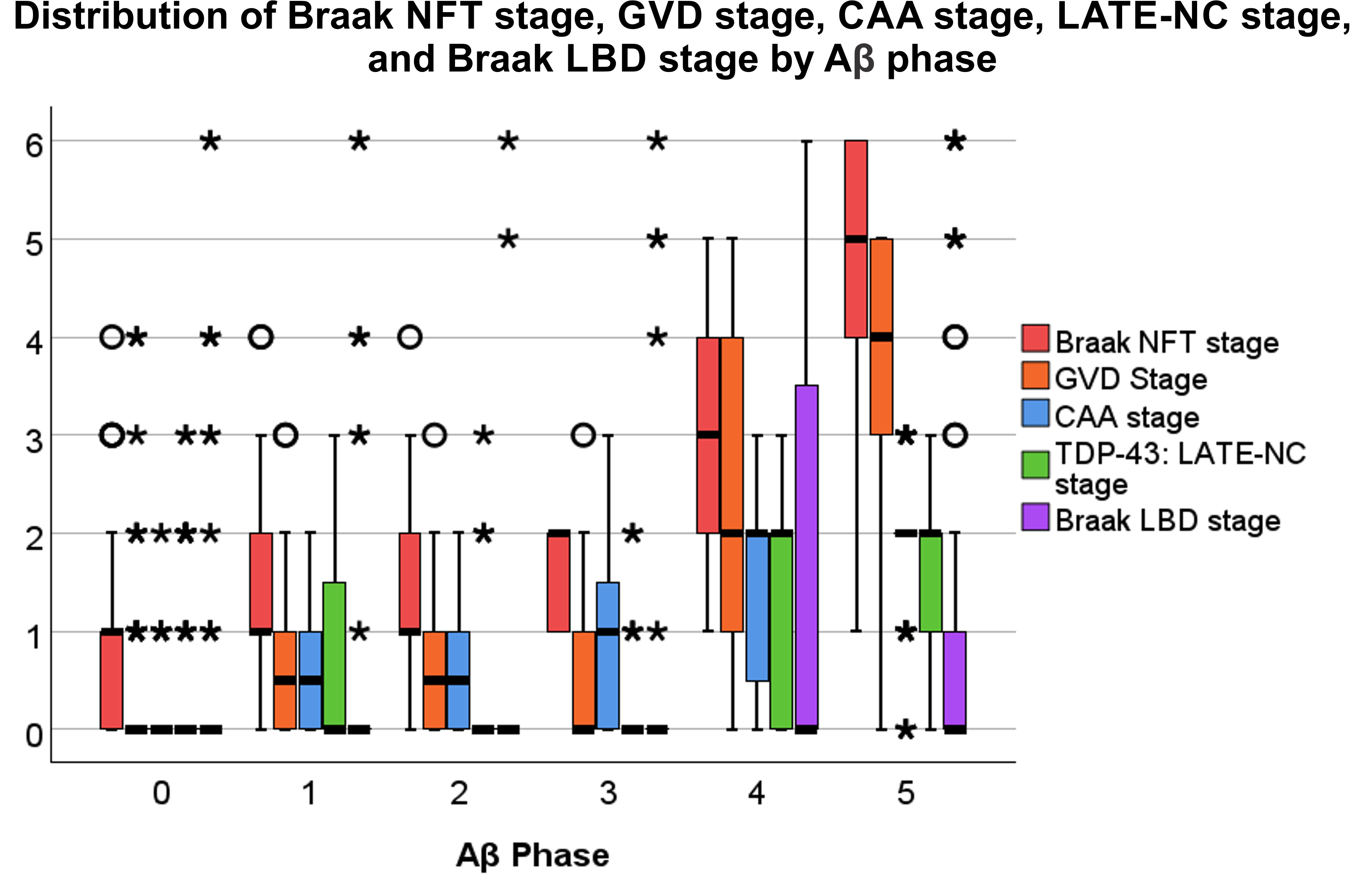


**Supplementary Figure 1**: Boxplot representation of the distribution of the Braak NFT stages (p-τ pathology) [1-3], GVD stages (necroptosis pathway activation) [4], CAA stages [5], LATE-NC stages (TDP-43 pathology) [6, 7], and Braak LBD stages (αSyn pathology) [8] with increasing Aβ plaque pathology represented by the phases of Aβ plaque deposition (Aβ phases) [9]. This figure provides the pathology distribution of the parameters for which only polynomial regression lines were depicted in Fig. 3a.

**Supplementary References**

1. Braak H, Alafuzoff I, Arzberger T, Kretzschmar H, Del Tredici K: **Staging of Alzheimer disease-associated neurofibrillary pathology using paraffin sections and immunocytochemistry.** *Acta Neuropathol* 2006, **112:**389-404.

2. Braak H, Braak E: **Neuropathological stageing of Alzheimer-related changes.** *Acta Neuropathol* 1991, **82:**239-259.

3. Braak H, Thal DR, Ghebremedhin E, Del Tredici K: **Stages of the pathologic process in Alzheimer disease: age categories from 1 to 100 years.** *J Neuropathol Exp Neurol* 2011, **70:**960-969.

4. Thal DR, Del Tredici K, Ludolph AC, Hoozemans JJ, Rozemuller AJ, Braak H, Knippschild U: **Stages of granulovacuolar degeneration: their relation to Alzheimer's disease and chronic stress response.** *Acta Neuropathol* 2011, **122:**577-589.

5. Thal DR, Ghebremedhin E, Orantes M, Wiestler OD: **Vascular pathology in Alzheimer disease: correlation of cerebral amyloid angiopathy and arteriosclerosis/lipohyalinosis with cognitive decline.** *J Neuropathol Exp Neurol* 2003, **62:**1287-1301.

6. Nelson PT, Dickson DW, Trojanowski JQ, Jack CR, Boyle PA, Arfanakis K, Rademakers R, Alafuzoff I, Attems J, Brayne C, et al: **Limbic-predominant age-related TDP-43 encephalopathy (LATE): consensus working group report.** *Brain* 2019, **142:**1503-1527.

7. Nelson PT, Lee EB, Cykowski MD, Alafuzoff I, Arfanakis K, Attems J, Brayne C, Corrada MM, Dugger BN, Flanagan ME, et al: **LATE-NC staging in routine neuropathologic diagnosis: an update.** *Acta Neuropathol* 2023, **145:**159-173.

8. Braak H, Del Tredici K, Rub U, de Vos RA, Jansen Steur EN, Braak E: **Staging of brain pathology related to sporadic Parkinson's disease.** *Neurobiol Aging* 2003, **24:**197-211.

9. Thal DR, Rub U, Orantes M, Braak H: **Phases of A beta-deposition in the human brain and its relevance for the development of AD.** *Neurology* 2002, **58:**1791-1800.
